# Anatomical dimensions of the lumbar dural sac predict the sensory block level of continuous epidural analgesia during labor

**DOI:** 10.1186/s12871-021-01485-5

**Published:** 2021-11-04

**Authors:** Chen-yang Xu, Can Liu, Xiao-ju Jin, Fan Yang, Fang Xu, Wan-Di Qian, Wen-jun Guo

**Affiliations:** 1 Department of Anesthesiology, the First Affiliated Hospital of Wannan Medical College, No. 2, Zheshan West Road, Jinghu District, Wuhu City, Anhui Province China; 2 Department of Anesthesiology, Women’s Hospital of Nanjing Medical University, Nanjing Maternity and Child Health Care Hospital, Nanjing, Jiangsu Province China

**Keywords:** Dural sac, Ultrasound, Labor analgesia, Continuous epidural anesthesia

## Abstract

**Background:**

The anatomical dimensions of the lumbar dural sac determine the sensory block level of spinal anesthesia; however, whether they show the same predictive value during continuous epidural anesthesia (CEA) remains undetermined. We designed the present study to verify the efficacy of the anatomical dimensions of the lumbar dural sac in predicting the sensory block level during labor analgesia.

**Methods:**

A total of 122 parturients with singleton pregnancies requesting labor analgesia were included in this study. The lumbar dural sac diameter (DSD), lumbar dural sac length (DSL), lumbar dural sac surface area (DSA), and lumbar dural sac volume (DSV) were measured with an ultrasound color Doppler diagnostic apparatus. CEA was performed at the L2-L3 interspace. After epidural cannulation, an electronic infusion pump containing 0.08% ropivacaine and sufentanil 0.4 μg/ml was connected. The sensory block level was determined with alcohol-soaked cotton, a cotton swab, and a pinprick. The analgesic efficacy of CEA was determined with a visual analog scale (VAS). The parturients were divided into two groups, “ideal analgesia” and “nonideal analgesia,” and the groups were compared by *t* test. Pearson’s correlation was performed to evaluate the association between the anatomical dimensions of the lumbar dural sac and sensory block level. Multiple linear regression analysis was used to create a model for predicting the sensory block level.

**Results:**

In the ideal analgesia group, the height, DSL, DSA, DSV and DSD were significantly smaller, and the body mass index (BMI) was significantly larger (*P* < 0.05). In addition, the DSL demonstrated the strongest correlation with the peak level of pain block (*r* = − 0.816, *P* < 0.0001; Fig. 2A), temperature block (*r* = − 0.874, *P* < 0.0001; Fig. 3A) and tactile block (*r* = − 0.727, *P* < 0.0001; Fig. 4A). Finally, the multiple linear regression analysis revealed that DSL and BMI contributed to predicting the peak sensory block level.

**Conclusion:**

In conclusion, our study shows that the sensory block level of CEA is higher when the DSL, DSA, DSV and DSD of puerperae are lower. DSL and BMI can be treated as predictors of the peak sensory block level in CEA during labor analgesia.

**Supplementary Information:**

The online version contains supplementary material available at 10.1186/s12871-021-01485-5.

## Background

Currently, continuous epidural anesthesia (CEA) is one of the preferred pain management methods for labor analgesia [1]. With the increasing requirements for the comfort of childbirth, the precision of anesthesia is increasingly required. Predicting the sensory block level of epidural anesthesia can provide a reference index for accurate perinatal anesthesia.

At present, it is widely believed that the effect of epidural anesthesia comes from delayed spinal anesthesia produced by local anesthetics in the epidural space that penetrate through the dura mater and penetrate into the cerebrospinal fluid (CSF) [2–4]. Fanning et al. reported that the length of the lumbar vertebrae had value in predicting drug diffusion in continuous combined spinal-epidural anesthesia [5]. In addition, the dural sac volume (DSV) affects the spread of local anesthetics in spinal anesthesia [5, 6]. As an important channel, the influence of the dura mater in epidural anesthesia is worthy of further study. Although ultrasound imaging of the lumbar spine cannot be used to determine the volume of CSF, it does allow the assessment of certain dimensions of the lumbar dural sac [7].

To verify whether these anatomical dimensions of the lumbar dural sac possess similar predictive value in determining the sensory block level in CEA during labor analgesia, we designed and performed this study using ultrasound.

## Methods

### Subjects

The study is conducted according to the principles of the Declaration of Helsinki and has been approved by the Chinese Ethics Committee of Registering Clinical Trials (ChiECRCT20200295). The study was registered in the Chinese Clinical Trial Registry on November 30th, 2019 (ChiCTR1900027830). This study was performed at the First Affiliated Hospital of Wannan Medical College and was conducted without any funding sources. From November 2019 to August 2020, a total of 122 parturients between the ages of 18 and 45 with an American Society of Anesthesiologists (ASA) status of II who received CEA analgesia for vaginal delivery were included in this clinical observational study. Written informed consent was obtained from all patients before participation. Patients with multiple pregnancies or a history of spinal anesthesia were excluded, as were patients who failed epidural puncture or switched to cesarean section without completing the study after enrollment. The dataset supporting the conclusions of this article is available upon request.

### Study protocol

**Fig. 1:**
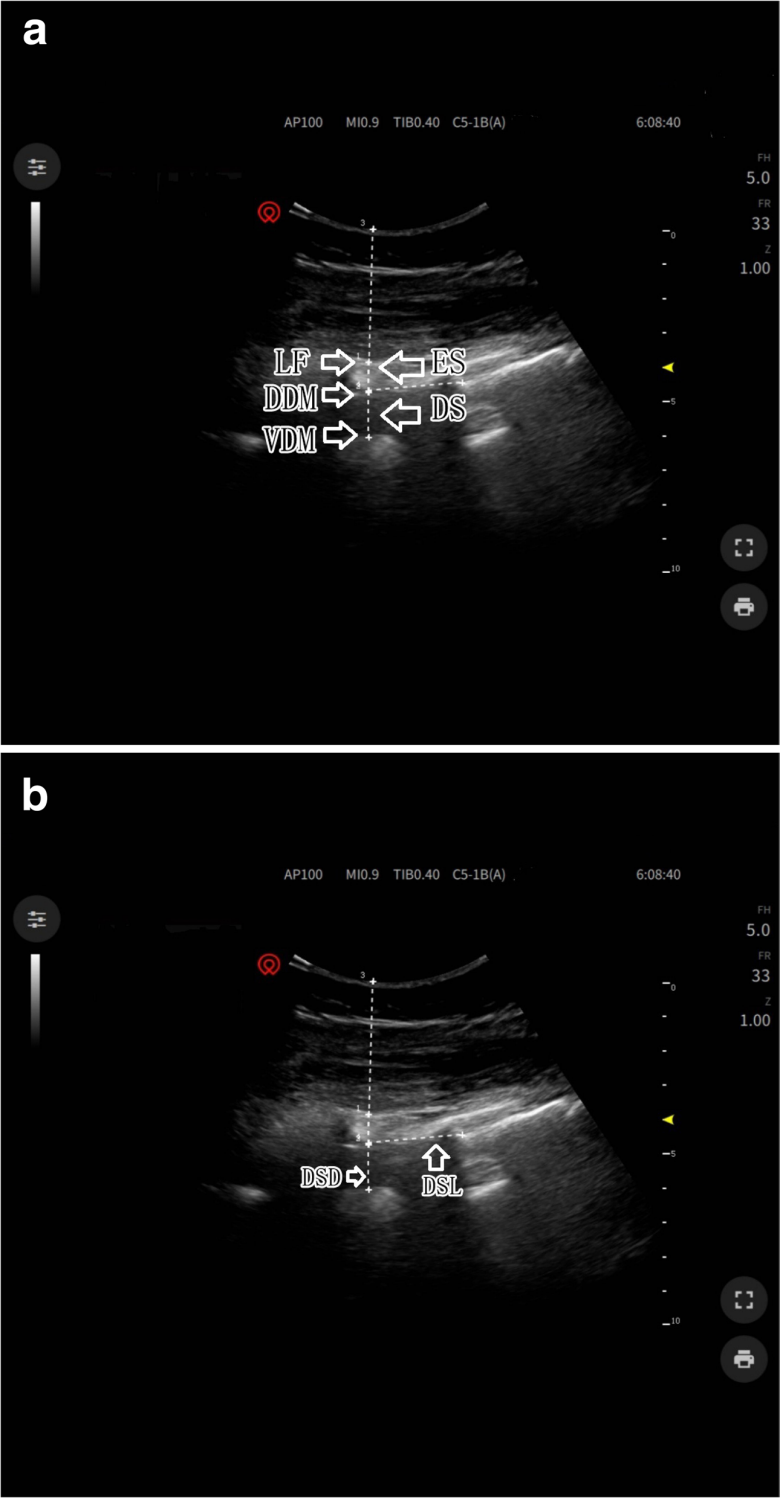
Measurement of the anatomical dimensions of the dural sac by ultrasound. **A** LF = ligamentum flavum, DDM = dorsal dura mater, VDM = ventral dura mater, ES = epidural space, DS = dural sac. **B** DSD = dural sac diameter, DSL = dural sac length

The parturients entered the operating room when the uterine opening was 3 cm, and routine monitoring was established. Before performing CEA, lactated Ringer’s solution was instilled for prehydration. Ultrasound scanning was performed before the administration of epidural analgesia via a portable ultrasound color Doppler diagnostic system equipped with a 2-5 MHz convex array probe (SonoScape Medical Corp., Shenzhen, China). Ultrasound imaging was performed with the patient lying on her left side, and the same position was used for epidural needle placement. In brief, an ultrasound probe was placed on the paramedian sagittal oblique plain to identify the L5-S1 interspace by identifying the continuous hyperechoic line of the sacrum. Then, the probe was slowly moved cephalad along the paramedian sagittal oblique plain to capture a view of the intervertebral space [8, 9]. Next, the L4-5, L3-4, L2-3, and L1-2 interspaces were determined in the same manner. The acoustic window included the vertebral body, ligamentum flavum, and dorsal and ventral dura mater (Fig. 1A). The distance from the dorsal dura mater to the ventral dura mater (lumbar dural sac diameter, DSD) was measured with a built-in caliper. The lumbar dural sac length (DSL) was defined as the sum of the each DSL between L1-2 and L5-S1 (Fig. 1B). The lumbar DSV and lumbar dural sac surface area (DSA) were obtained by adding the DSV and DSA, respectively, between each lumbar intervertebral space.

In the past, the lumbar dural sac was assumed to be cylindrical [5]. In view of the fact that some scholars have found that the diameter of the dural sac is different in different intervertebral spaces [10], the formula for a circular truncated cone was applied to calculate the volume and surface area of the lumbar dural sac in our study. The volume of each dural sac was calculated according to the formula for the volume of a circular truncated cone: *V* = *πh*(*R*^*2*^ + *r*^*2*^ + *Rr*)/*3*. The surface area of each dural sac was calculated according to the formula for the surface area of a circular truncated cone: *S* = *πRl* + *πrl*, where the radius is half of the DSD, h is the DSL, and l is calculated from r and h by $l=\sqrt{{\left(R-r\right)}^2+{h}^2}$.

Cross-sectional imaging was carried out in the L2-3 intervertebral space to determine the ideal puncture point at the midpoint of L2-3 intervertebral space [11]. We also measured the depth of the epidural space and the distance between the midpoints of the T12-L1 and L2-3 intervertebral spaces. The sum of the two lengths was used to determine the depth of the epidural catheter placement. This was done to ensure that the opening of the catheter tip was located at the midpoint of the T12-L1 intervertebral space.

With the parturient in a supine position, 3 ml of 1.5% lidocaine was infused as an experimental dose to rule out the risk of spinal anesthesia. Then, an electronic infusion pump (APON Corporation, Nantong, China) containing sufentanil 0.4 μg/ml and 0.08% ropivacaine was connected to the epidural catheter. The initial dose was 8 ml administered at a rate of 1 ml/s, and continuous infusion was administered at a rate of 8 ml/h until the uterine orifice was fully opened. During the whole procedure, 6 mg ephedrine was administered intravenously when the post-anesthesia systolic blood pressure decreased by more than 20%, and 0.2 mg atropine was injected when the heart rate was below 55 beats per minute. In patients presenting a risk of spinal anesthesia, epidural analgesia was suspendend and if necessary, appropriate rescue medical care was initiated.

The efficacy of labor analgesia was evaluated with a visual analog scale (VAS) at time zero, which was just after epidural cannulation. The level of pain, temperature and tactile sensory block were tested with pinprick, alcohol-soaked cotton and cotton swabs, respectively. Evaluations of the sensory block level and VAS score were performed every minute within the first 3 min and every 5 min after administration of the initial dose. After three consecutive evaluation values remained unchanged, the sensory block level and VAS score were tested every 30 min until the end of labor.

“Ideal analgesia” was defined as “a VAS score decline to 3 points within 30 minutes” [12, 13]. If the maternal VAS score did not reach 3 points within 30 min, it was regarded as “nonideal analgesia.” If her sensory block level was fixed (i.e., the same value for three consecutive assessments), an additional 8 ml of the drug was added using an electronic infusion pump.

### Statistical analysis

G-power 3.1.9.2 was used to calculate the sample size. In this study, five predictors were included in a multiple linear regression analysis, including maternal body mass index (BMI), DSD, DSL, DSA and DSV. The expected effect value was 0.15, the test level was 0.05, and the test power was 0.9, so the minimum sample size was 118.

The following software was used for analysis: Excel 2010, GraphPad Prism 8.0.1, and SPSS software (version 25.0, SPSS Inc., Chicago, IL, USA). The results are presented as the mean ± standard deviation (SD), and comparisons between groups were performed using unpaired Student’s *t* test or Welch’s *t* test. The sensory block level is expressed as the median and range. Correlations between patient characteristics and the sensory block level were analyzed via Pearson’s correlation. Multiple linear regression analysis was used to analyze the five explanatory variables of BMI, DSL, DSA, DSV and DSD and the sensory block level. The prediction model with the highest adjusted *R*^*2*^ value was selected by a stepwise method. Due to collinearity (the DSA and DSV were calculated using the DSL as part of the formula), the DSA and DSV could not appear in the same model when constructing predictive models. Statistical significance was defined as *P* < 0.05 (two-sided).

## Results

Three parturients transferred to cesarean section, and the remaining 119 parturients completed the study and were included in the analysis (Supplementary Data 1, Supplementary Tables 1-2). As previously described, we classified parturients who underwent epidural analgesia into two groups: ideal analgesia [14] and nonideal analgesia [15]. Between the two groups, the height, DSL, DSA, DSV and DSD of the ideal analgesia group were significantly smaller, and the BMI was significantly larger (*P* < 0.05) (Table 1).

**Table 1:** Comparison between the ideal analgesia group and the nonideal analgesia group (*n* = 119)

Patient characteristics	Ideal analgesia (*n* = 87)	Nonideal analgesia (*n* = 32)	*P*
Height, cm	160.58 ± 4.45	164.94 ± 3.56	< 0.0001
Weight, kg	69.52 ± 8.45	66.56 ± 8.58	0.094
BMI, kg/m^2^	26.98 ± 2.89	24.36 ± 2.69	< 0.0001
DSL, cm	11.31 ± 1.17	13.34 ± 1.17	< 0.0001
DSA, cm^2^	49.01 ± 7.40	62.20 ± 10.96	< 0.0001
DSV, cm^3^	17.12 ± 3.95	23.65 ± 7.37	< 0.0001
DSD, cm	1.36 ± 0.12	1.45 ± 0.20	0.016

**Fig. 2:**
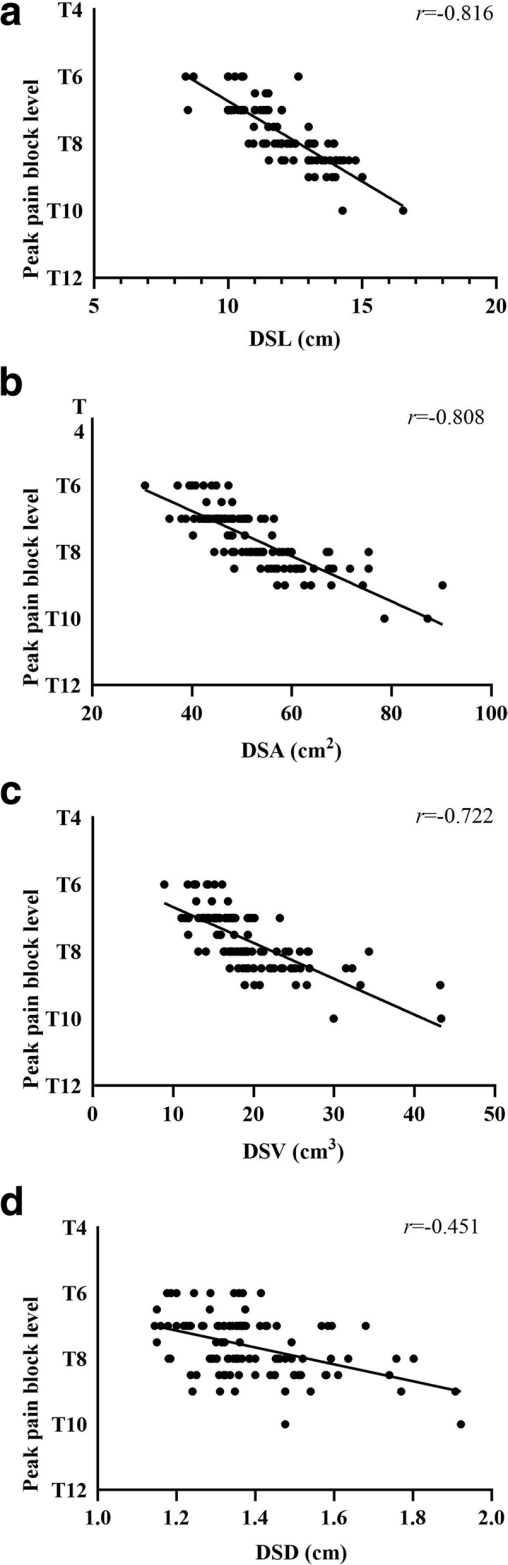
**A** Correlation between the lumbar dural sac length (DSL) and peak pain block level (*r* = − 0.816, *p* < 0.0001). **B** Correlation between the lumbar dural sac surface area (DSA) and peak pain block level (*r* = − 0.808, *p* < 0.0001). **C** Correlation between the lumbar dural sac volume (DSV) and peak pain block level (*r* = − 0.722, *p* < 0.0001). **D** Correlation between the lumbar dural sac diameter (DSD) and peak pain block level (*r* = − 0.451, *p* < 0.0001). Although correlation coefficients (*r*) and *P* values were calculated using Pearson’s correlation, the linear regression lines are presented in these graphs

**Fig. 3:**
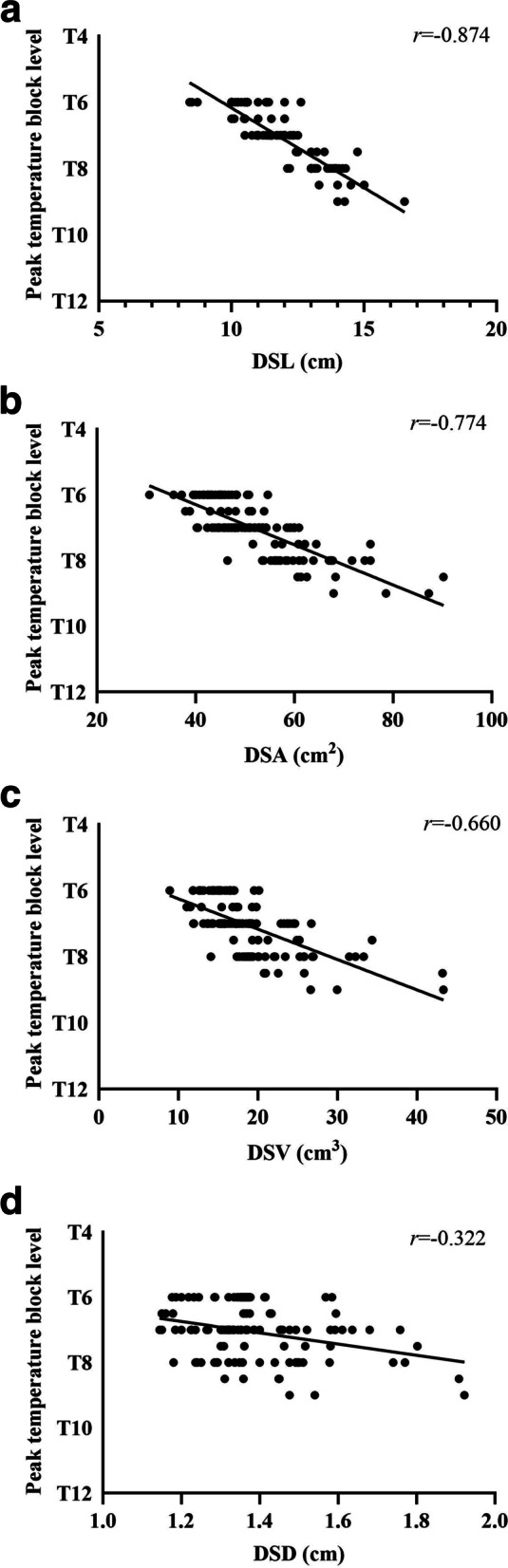
**A** Correlation between the lumbar dural sac length (DSL) and peak temperature block level (*r* = − 0.874, *p* < 0.0001). **B** Correlation between the lumbar dural sac surface area (DSA) and peak temperature block level (*r* = − 0.774, *p* < 0.0001). **C** Correlation between the lumbar dural sac volume (DSV) and peak temperature block level (*r* = − 0.66, *p* < 0.0001). **D** Correlation between the lumbar dural sac diameter (DSD) and peak temperature block level (*r* = − 0.322, *p* < 0.0001). Although correlation coefficients (*r*) and *P* values were calculated using Pearson’s correlation, the linear regression lines are presented in these graphs

**Fig. 4:**
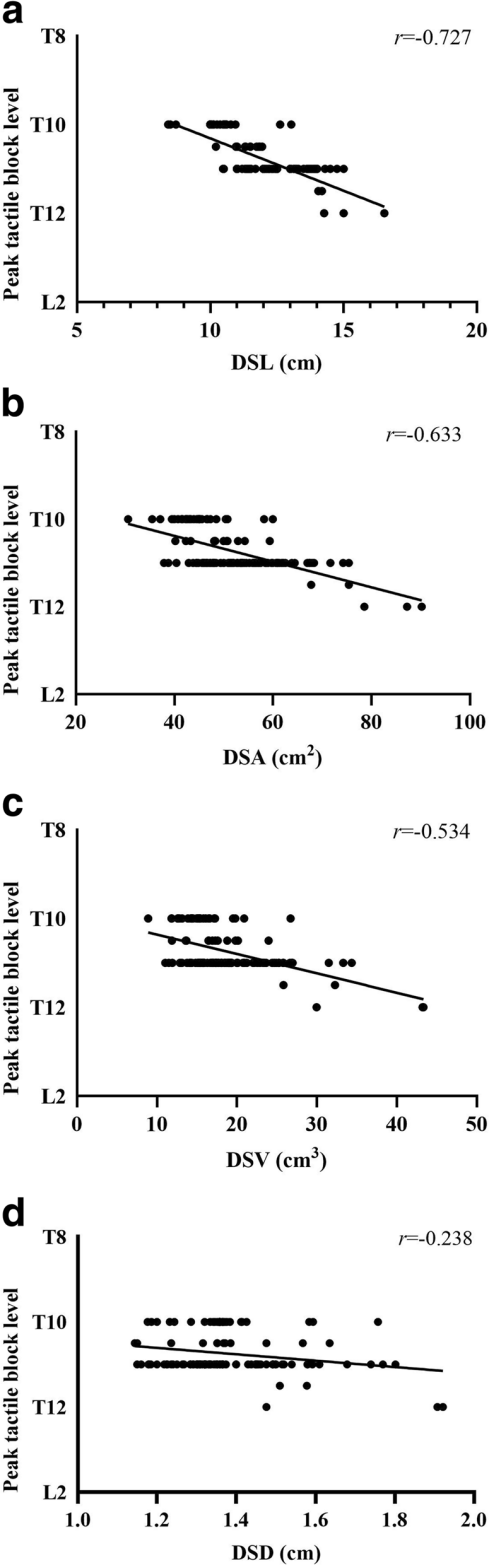
**A** Correlation between the lumbar dural sac length (DSL) and peak tactile block level (*r* = − 0.727, *p* < 0.0001). **B** Correlation between the lumbar dural sac surface area (DSA) and peak tactile block level (*r* = − 0.633, *p* < 0.0001). **C** Correlation between the lumbar dural sac volume (DSV) and peak tactile block level (*r* = − 0.534, *p* < 0.0001). **D** Correlation between the lumbar dural sac diameter (DSD) and peak tactile block level (*r* = − 0.238, *p* < 0.0001). Although correlation coefficients (*r*) and *P* values were calculated using Pearson’s correlation, the linear regression lines are presented in these graphs

Pearson’s correlation demonstrated a correlation between the DSL, DSA, DSV and DSD and the level of pain, temperature and tactile sensory block (Figs. 2, 3, 4, Supplementary Tables 3, 4, 5). The DSL demonstrated the strongest correlation with the peak level of pain block (*r* = − 0.816, *P* < 0.0001; Fig. 2A), temperature block (*r* = − 0.874, *P* < 0.0001; Fig. 3A) and tactile block (*r* = − 0.727, *P* < 0.0001; Fig. 4A).

DSL and BMI were important predictors of the peak sensory block level. The multiple linear regression analysis revealed the following (Table 2):

$$\mathrm{Peak}\ \mathrm{pain}\ \mathrm{block}\ \mathrm{level}=4.7+0.452\times \mathrm{DSL}-0.093\times \mathrm{BMI}$$

$$\mathrm{Peak}\ \mathrm{temperature}\ \mathrm{block}\ \mathrm{level}=3.409+0.461\times \mathrm{DSL}-0.069\times \mathrm{BMI}$$

$$\mathrm{Peak}\ \mathrm{tactile}\ \mathrm{block}\ \mathrm{level}=9.505+0.220\times \mathrm{DSL}-0.052\times \mathrm{BMI}$$

**Table 2:** Multiple Linear Regression Models

Response Variable	*R*^*2*^	Adjusted *R*^*2*^	Intercept	*P*	Explanatory Variables	Regression Coefficient	Standard Regression Coefficient
Peak pain block level	0.771	0.767	4.700	< 0.0001 < 0.0001	DSL BMI	0.452 − 0.093	0.769 − 0.328
Peak temperature block level	0.829	0.826	3.409	< 0.0001	DSL	0.461	0.836
				< 0.0001	BMI	−0.069	− 0.260
Peak tactile block level	0.638	0.632	9.505	< 0.0001 < 0.0001	DSL BMI	0.220 −0.052	0.679 −0.334

## Discussion

In this study, we first observed a smaller DSL, DSA, DSV and DSD in patients with ideal analgesia. In addition, our results suggested that there is a negative correlation between the peak sensory block level (pain, temperature and tactile) and the anatomical dimensions of the lumbar dural sac (DSL, DSA and DSV). Finally, multiple linear regression analysis revealed that DSL and BMI contributed to predicting the peak sensory block level.

The sensory block level in CEA determines the efficacy of analgesia, which is the most concerning issue for parturients during labor. However, the sensory block level is affected by a variety of factors, such as the operating proficiency, insertion depth, injection rate, and drug concentration. Previous studies have used many anatomical variables to explain the diffusion of local anesthetics in CSF, such as height, weight [13], spine length [16], and abdominal girth [17].

The epidural space between the dura mater and the vertebral canal wall is used as a route for administering local anesthetics. The spreading of local anesthetics into the epidural space after injection involves two steps [2]. First, local anesthetics spread within the epidural space itself. This is dependent on the conditions that have been previously discussed, such as the dose, volume and infusion rate of local anesthetics. Second, local anesthetics penetrate into the subperineural space by spreading around the capillary and lymphatic channels of the vasa nervorum at the dura mater [2, 3]. Previous experiments have shown that the dose of drugs that reach the subpial spaces around the spinal cord and can diffuse along the nerve axis will be proportional to the dose that can spread through the dura mater into the subperineural space [2]. Ultimately, most studies have indicated that local anesthetics penetrate through the dura mater and spread in the CSF after epidural injection, which produces delayed spinal anesthesia [2–4]. Carpenter and colleagues described that a smaller volume of CSF leads to a greater sensory block level in spinal anesthesia [18]. Thus, the volume of CSF is an important anatomical factor affecting the sensory block level of epidural anesthesia.

Previous studies have shown that obese patients under spinal anesthesia have a higher level of sensory block [19], which may be due to the narrowing of the epidural space and increased epidural pressure caused by dilated epidural veins and accumulated epidural fat in these patients [20]. Compression of the lumbar dural sac causes a reduction in the volume of cerebrospinal fluid in the waist, reducing the dilution of local anesthetics [21]. Parturients are a special type of abdominal obesity patient [22]. Given that maternal epidural fat is difficult to accurately display under ultrasound, BMI was included as an indicator of obesity in our study.

Based on previous studies showing that the anatomical dimensions of the lumbar dural sac can be measured to assess the volume of CSF [5, 6], we selected DSL, DSA, DSV and DSD as the independent variables. In our study, the DSL, DSA, DSV and DSD were significantly lower in patients with ideal analgesia. To identify the factor with the strongest correlation at the sensory block level, we performed Pearson’s correlation analysis. The DSL, DSA, DSV and DSD displayed negative correlations with the level of pain, temperature and tactile sensory block. In our study, the correlation between height and sensory block level was relatively small, while the correlation between DSL and sensory block level was higher. Because the height differences between most adults are determined by the length of the long bones in the lower limbs rather than the length of the spine, the measurement of DSL has more clinical application value. Our DSA measured by ultrasound has a similar correlation with the DSA measured by Higuchi et al. using magnetic resonance imaging (MRI) [23]. In addition, our results break through the noncorrelation between DSD and the sensory block level in spinal anesthesia [24], proving that there is a negative correlation between DSD and the sensory block level in epidural anesthesia, which provides a new idea for the clinical study of intraspinal anesthesia.

There are several limitations to this study. The volume of fluid injected into the epidural space compresses the dural sac and reduces the volume of CSF [25]. Although we limited the volume of the experimental dose of lidocaine to 3 ml, we cannot ignore the effect on the sensory block level. In addition, ultrasound imaging cannot display soft tissue, such as fat [26] or vascular tissue, in the epidural space clearly and accurately [27]; thus, we should not rule out the influence of soft tissues on the current results. Furthermore, although our dural sac model was based on the formula for a circular truncated cone, the values were approximated.

Our study provides a convenient and noninvasive method to predict the efficacy of labor analgesia in parturients. In addition, with this method, we can screen patients with a high risk of nonideal analgesia to adjust the dose, volume and infusion rate of local anesthetics. Further studies at multiple centers with larger populations are necessary to explore the suitable drug dose, volume and infusion rate and to provide a reference index for accurate perinatal anesthesia.

## Conclusions

In conclusion, our study shows that the sensory block level of CEA is higher when the DSL, DSA, DSV and DSD of puerperae are lower. DSL and BMI can be treated as predictors of the peak sensory block level in CEA during labor analgesia.

## Supplementary Information

**Additional file 1.** Supplementary Data 1.

**Additional file 2: Supplemental Table 1.** Patient characteristics (*n* = 119).

**Additional file 3: Supplemental Table 2.** Sensory block levels.

**Additional file 4: Supplemental Table 3.** Correlations between patient characteristics and pain block level.

**Additional file 5: Supplemental Table 4.** Correlations between patient characteristics and temperature block level.

**Additional file 6: Supplemental Table 5.** Correlations between patient characteristics and tactile block level.

## Data Availability

The datasets generated and/or analysed during the current study are not publicly available due to limitations of ethical approval involving the patient data and anonymity but are available from the corresponding author on reasonable request.
