# Supplementary material for: Anatomical dimensions of the lumbar dural sac predict the sensory block level of continuous epidural analgesia during labor
Source: BMC Anesthesiol. 2021 Nov 4;21:268. doi: 10.1186/s12871-021-01485-5 (PMC8567596; doi:10.1186/s12871-021-01485-5)
Supplement: Supplementary file 2 — Additional file 2: Supplemental Table 1. Patient characteristics (n = 119). [file 12871_2021_1485_MOESM2_ESM.docx]

Supplemental Table 1. Patient characteristics (n=119)

| Characteristics |  | Mean±SD |
| --- | --- | --- |
| Age, y  Height, cm  Weight, kg  BMI, kg/m^2^  DSL, cm  DSA, cm^2^  DSV, cm^3^  DSD, cm |  | 27.26±3.00  161.69±4.68  68.90±8.51  26.37±3.06  11.84±1.48  52.47±10.33  18.84±5.85  1.39±0.15 |
