# Supplementary material for: Anatomical dimensions of the lumbar dural sac predict the sensory block level of continuous epidural analgesia during labor
Source: BMC Anesthesiol. 2021 Nov 4;21:268. doi: 10.1186/s12871-021-01485-5 (PMC8567596; doi:10.1186/s12871-021-01485-5)
Supplement: Supplementary file 3 — Additional file 3: Supplemental Table 2. Sensory block levels. [file 12871_2021_1485_MOESM3_ESM.docx]

Supplemental Table 2. Sensory block levels

| Sensory block levels | \| Median \| \| --- \| | Range |
| --- | --- | --- | --- |
| \| Pain block level at 30 min  Peak level of pain block  Temperature block level at 30 min  Peak level of temperature block  Tactile block level at 30 min  Peak level of tactile block \| \| --- \| | \| T8 \| \| --- \| \| T7.5 \| \| T7  T7  T11 \| \| T11 \| | \| \| T10-T6 \| \| --- \| \| T10-T6 \| \| T9-T6  T9-T6  T12-T10 \| \| T12-T10 \| \| \| --- \| --- \| --- \| --- \| --- \| |

T7.5=Between T7 and T8
