# Supplementary material for: Anatomical dimensions of the lumbar dural sac predict the sensory block level of continuous epidural analgesia during labor
Source: BMC Anesthesiol. 2021 Nov 4;21:268. doi: 10.1186/s12871-021-01485-5 (PMC8567596; doi:10.1186/s12871-021-01485-5)
Supplement: Supplementary file 4 — Additional file 4: Supplemental Table 3. Correlations between patient characteristics and pain block level. [file 12871_2021_1485_MOESM4_ESM.docx]

Supplemental Table 3. Correlations between patient characteristics and pain block level

| Characteristics | 30 min | | Peak | |
| --- | --- | --- | --- | --- |
|  | *r* | *P* | *r* | *P* |
| Height, cm | -0.544 | <0.0001 | -0.532 | <0.0001 |
| Weight, kg | 0.141 | 0.127 | 0.139 | 0.132 |
| BMI, kg/m^2^ | 0.446 | <0.0001 | 0.438 | <0.0001 |
| DSL, cm | -0.812 | <0.0001 | -0.816 | <0.0001 |
| DSA, cm^2^ | -0.806 | <0.0001 | -0.808 | <0.0001 |
| DSV, cm^3^ | -0.723 | <0.0001 | -0.722 | <0.0001 |
| DSD, cm | -0.466 | <0.0001 | -0.451 | <0.0001 |
