# Supplementary material for: Anatomical dimensions of the lumbar dural sac predict the sensory block level of continuous epidural analgesia during labor
Source: BMC Anesthesiol. 2021 Nov 4;21:268. doi: 10.1186/s12871-021-01485-5 (PMC8567596; doi:10.1186/s12871-021-01485-5)
Supplement: Supplementary file 5 — Additional file 5: Supplemental Table 4. Correlations between patient characteristics and temperature block level. [file 12871_2021_1485_MOESM5_ESM.docx]

Supplemental Table 4. Correlations between patient characteristics and temperature block level

| Characteristics | 30 min | | Peak | |
| --- | --- | --- | --- | --- |
|  | *r* | *P* | *r* | *P* |
| Height, cm | -0.550 | <0.0001 | -0.555 | <0.0001 |
| Weight, kg | 0.070 | 0.447 | 0.066 | 0.473 |
| BMI, kg/m^2^ | 0.380 | <0.0001 | 0.380 | <0.0001 |
| DSL, cm | -0.866 | <0.0001 | -0.874 | <0.0001 |
| DSA, cm^2^ | -0.772 | <0.0001 | -0.774 | <0.0001 |
| DSV, cm^3^ | -0.661 | <0.0001 | -0.660 | <0.0001 |
| DSD, cm | -0.326 | <0.0001 | -0.322 | <0.0001 |
