# Supplementary material for: Anatomical dimensions of the lumbar dural sac predict the sensory block level of continuous epidural analgesia during labor
Source: BMC Anesthesiol. 2021 Nov 4;21:268. doi: 10.1186/s12871-021-01485-5 (PMC8567596; doi:10.1186/s12871-021-01485-5)
Supplement: Supplementary file 6 — Additional file 6: Supplemental Table 5. Correlations between patient characteristics and tactile block level. [file 12871_2021_1485_MOESM6_ESM.docx]

Supplemental Table 5. Correlations between patient characteristics and tactile block level

| Characteristics | 30 min | | Peak | |
| --- | --- | --- | --- | --- |
|  | *r* | *P* | *r* | *P* |
| Height, cm | -0.500 | <0.0001 | -0.508 | <0.0001 |
| Weight, kg | 0.181 | 0.049 | 0.140 | 0.128 |
| BMI, kg/m^2^ | 0.460 | <0.0001 | 0.431 | <0.0001 |
| DSL, cm | -0.713 | <0.0001 | -0.727 | <0.0001 |
| DSA, cm^2^ | -0.634 | <0.0001 | -0.633 | <0.0001 |
| DSV, cm^3^ | -0.536 | <0.0001 | -0.534 | <0.0001 |
| DSD, cm | -0.254 | <0.0001 | -0.238 | <0.0001 |
